# Fecal Scrotal Abscess Secondary to Spontaneous Retroperitoneal Perforation of Ascending Colon

**DOI:** 10.1155/2021/6658083

**Published:** 2021-03-29

**Authors:** Akshay Bahadur, Nirmala Singh, Mayank Kashmira, Ashish Shukla, Vikas Gupta, Shashank Jain

**Affiliations:** ^1^Department of General Surgery, Dr. Hedgewar Arogya Sansthan, Delhi, India; ^2^VVWC–CGHS, Delhi, India; ^3^Nishkam Imaging Solution Pvt. Ltd., Delhi, India

## Abstract

**Introduction:**

Fecal abscess or enterocutaneous fistulas of the scrotum are rare and are invariably the result of incarcerated bowel loop in inguinal hernia. Spontaneous perforation of the colon (SPC) having no definite cause is also rare. Much rarer is posterior colonic perforations causing an extensively large retroperitoneal abscess. Similarly, spread of retroperitoneal abscess to the thigh or scrotum has rarely been reported. We report a case of spontaneous posterior perforation of ascending colon resulting in large retroperitoneal abscess eventually causing scrotal abscess, which resolved on conservative treatment and drainage of the scrotal fecal abscess. *Case Presentation*. A 20-year-old male presented with gradually increasing noncolicky pain right side abdomen with nonprojectile vomiting, obstipation, and progressive abdominal distension. Clinically, the abdomen was tender with guarding over the right side with signs of inflammation on the right side back with no associated hernia. On conservative treatment, he was gradually improved but developed right side scrotal abscess a week later. CT abdomen showed a large retroperitoneal collection having multiple internal air lucencies, displacing ascending colon and caecum medically with discontinuity in the posterior wall of ascending colon. The large retroperitoneal collection was extending from right pararenal and posterior perihepatic soft tissue planes to the right iliac fossa and thigh. On drainage of the scrotal abscess, about 350 ml of fecal contents was evacuated. The patient gradually recovered and was discharged on conservative treatment with an uneventful 4-year follow-up.

**Conclusion:**

Diagnosis of retroperitoneal perforation of the colon is often delayed due to the absence of peritoneal irritation. An extensively large retroperitoneal abscess may spread the infection to the scrotum and thigh due to extreme pressure, possibly by dissecting away the transversalis fascia through a deep ring along the side of the spermatic cord. Timely performed CT/MRI can avoid delay in the diagnosis of retroperitoneal abscess and further spread of infection.

## 1. Introduction

Enterocutaneous fistula of the scrotum is rare [1] and is almost exclusively due to incarcerated bowel loop in inguinal hernia. Spontaneous perforation of the colon (SPC) is rare [2, 3] and is defined as perforation of a normal colon without any contributing factor such as disease of the bowel or hernia [3]. The retroperitoneal colonic perforations are rare causes of a retroperitoneal abscess and are exclusively seen in frail elderly patients [4]. No case of spontaneous retroperitoneal perforation of ascending colon causing an abscess or enterocutaneous fistula of the scrotum in the absence of hernia has been ever reported to the best of our knowledge in indexed literature. We describe our unique case of a 20-year-old male with spontaneous retroperitoneal perforation of ascending colon resulting in initially retroperitoneal abscess, later converting to scrotal fecal abscess, managed with conservative treatment and drainage of the abscess.

## 2. Case Presentation

A 20-year-old male presented to the emergency department complaining of gradually increasing noncolicky pain right side abdomen with multiple episodes of nonprojectile vomiting, obstipation, and progressive abdominal distension for the past 3 days. There was fever with chills and rigor for a similar duration. There was no past history of chronic constipation. There was no personal or family history of *tuberculosis*. At the time of presentation, the patient was having a pulse rate of 110 beats/minute, a blood pressure of 120/82 mm Hg, and a temperature of 39.5° Celsius. During the abdominal examination, there was tenderness and guarding over the right side of the abdomen with signs of inflammation on the right side back. There was no associated inguinal hernia. On auscultation, bowel sounds were sluggish.

Blood investigations revealed haemoglobin of 11 gm/dL, white cell count of 10,900/cumm with 80% polymorphs, 16% lymphocytes, 2% monocytes, and 2% eosinophil. Liver function test, renal function test, serum electrolyte, serum glucose, and urine analysis were all normal. Typhi antigen card test was negative.

Abdominal X-ray in the erect position showed the right side minimal pleural effusions with dilated loops of the small bowel. Ultrasonography (USG) of the abdomen reported multiple fluid distended intestinal loops suspecting paralytic ileus/subacute intestinal obstruction. The patient was kept on conservative treatment with Ryle's tube suction, intravenous fluids, and parenteral antibiotics. By the 4^th^ day of conservative treatment, the patient started passing flatus and feces and was gradually shifted to a liquid diet.

**Figure 1:**
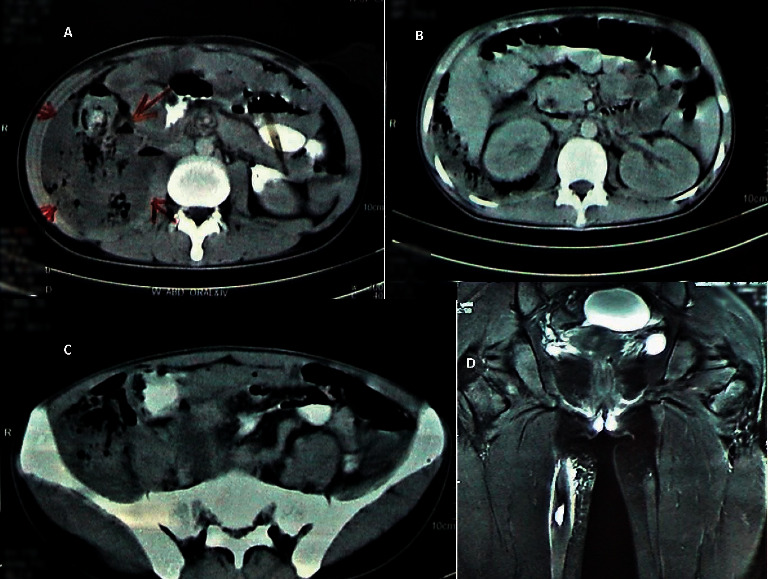
CT scan abdomen with oral contrast showed a large retroperitoneal collection with multiple air lucencies displacing ascending colon and caecum medially. (a) Discontinuity in the posterior wall of ascending colon with a large collection having internal air lucencies, (b) collection with internal air lucencies seen in the right pararenal and posterior perihepatic soft tissue planes, (c) retroperitoneal collection seen tracking into the right iliac fossa, and (d) elongated collection and surrounding soft tissue oedema seen along hamstring muscles of the right thigh.

On the 8^th^ day of admission, the patient complained of painful swelling right scrotum. Scrotal examination showed signs of inflammation with fluctuation on the right side. CT scan of the abdomen showed discontinuity in the posterior wall of ascending colon with a large retroperitoneal collection having multiple internal air lucencies, displacing ascending colon and caecum medically. The right retroperitoneal collection extending superiorly from right pararenal and posterior perihepatic soft tissue planes to the right iliac fossa and right thigh (Figure 1).

**Figure 2:**
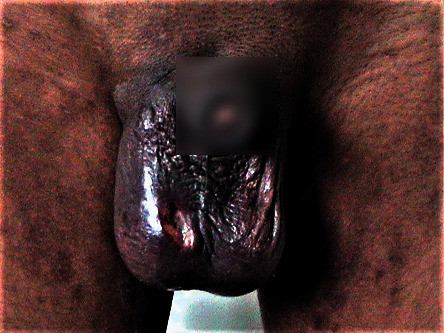
Scrotal wound 5^th^ day following drainage of fecal contents.

Around 350 ml of fecal contents was evacuated by incision & drainage (I&D). The fecal discharge gradually started decreasing while the patient was continued on a liquid diet (Figure 2). The cellulitis of the right side back was also started decreasing. On the 26th day of admission, the patient was discharged in satisfactory condition with no discharge from the scrotal wound. 4-year follow-up was uneventful.

## 3. Discussion

Almost all fecal abscesses/enterocutaneous fistulas of the scrotum are due to incarcerated bowel loop in inguinal hernia. Paediatric age is the prominent group for these abscess/fistula, while adults are comparatively spared [5–7].

After extensive research of indexed literature, we find 33 cases of enterocutaneous fistula/fecal abscess of the scrotum, labia, or inguinal region. In our review, we found that the fecal fistulas/abscesses were either present in below 40 days of age (13 cases, mostly neonates) or above the age of 20 years (17 cases, mostly above 40 years of age) (Table 1). The inguinal hernia was present in all cases except ours, where there was no hernia present. All except two (who refused to operate) were managed by exploratory laparotomy and anastomosis of disrupted bowel, while our case was managed by conservative treatment along with incision & drainage of the scrotal fecal abscess.

Spontaneous perforation of the colon (SPC) is a perforation of the normal colon in the absence of a pathological cause such as tumours, diverticulosis, or external injury [33]. Colonic perforations are mostly encountered in diverticulitis, carcinoma colon, inflammatory bowel disease, trauma, foreign body insertion, and iatrogenic [34]. The cause of spontaneous colonic perforation is unclear. Hard feces present in patients with chronic constipation compress the colonic wall resulting in diminished blood supply, which may lead to significant feculent ulcer following ischemia and necrosis of colonic mucosa [35].

The posterior colonic perforations are rare causes of retroperitoneal abscess and are relatively seen in frail elderly patients [4]. The retroperitoneum, a potential space with clearly defined boundaries between the peritoneum and the transversalis fascia, can be seeded by infections involving surrounding organs such as kidneys, pancreas, colon, duodenum, bladder, uterus, and rectum [36]. Perforations of bowel in retroperitoneal spaces usually presented with unspecific symptomatology [37], where pyrexia of unknown origin is a common presentation. Less commonly, it may present with pain in the lower back, hip, or thigh. Other symptoms that may be present include malaise, anorexia, and weight loss [38], or painful inguinal swellings [39].

Though it is not the common course, retroperitoneal abscesses may rarely extend to the thigh or scrotum, and such abscesses may be missed to diagnosed for as long as two weeks from the onset of abdominal pain [40]. Infection and air that has developed in retroperitoneal space due to pathology of surrounding organs are contained by transversalis fascia but may rarely dissect away through a deep inguinal ring alongside the spermatic cord in males and the round ligament in women, respectively, to reach scrotum and grand labia when there is tremendous pressure due to their massive size [41, 42]. The pathophysiological mechanism involved is the emergence of a pressure gradient between the peritoneum and surrounding structures, causing rupture of the perianal tissue, allowing gas from a perforation to diffuse along tissue planes [43]. In our case also, the retroperitoneal abscess was very extensive, occupying almost all possible retroperitoneal space of the right side extending to the thigh and ipsilateral scrotum.

The diagnostic sensitivity of ultrasonography for retroperitoneal abscesses is 67%–87%. CT has a sensitivity ranging from 90% to 100%, while MRI has a sensitivity ranging from 88.5% to 100% in diagnosing retroperitoneal abscesses, and both are reliable investigations [44].

Further, we found only 12 cases of spontaneous perforation of the colon (SPC) but no case of spontaneous retroperitoneal perforation of ascending colon, or any case of fecal abscess/enterocutaneous fistula of the scrotum in the absence of inguinal hernia and no case of retroperitoneal fecal abscess reaching scrotum in indexed literature. Spontaneous retroperitoneal perforation of ascending colon resulting in retroperitoneal abscess and eventually ending into scrotal fecal abscess in the absence of inguinal hernia was albeit a surprisingly rare case reported in the literature.

## 4. Conclusion

Pneumoscrotum and fecal abscess of the scrotum are an extremely rare presentation of retroperitoneal colonic perforation, especially in the absence of inguinal hernia. Diagnosis of retroperitoneal perforation of the colon is often delayed due to the absence of peritoneal irritation. Our experience suggests that, due to extreme pressure, an extensively large retroperitoneal abscess may spread the infection to the scrotum and thigh possibly by dissecting away the transversalis fascia through a deep ring along the side of the spermatic cord. CT scan or MRI performed in undiagnosed case of the acute abdomen can avoid delay in the diagnosis of a retroperitoneal abscess, as well as further spread of infection.

## Figures and Tables

**Table 1:** Review of literature of enterocutaneous fistula (ECF)/fecal abscess of the scrotum.

S. no.	Author	Age (years)	Sex	Clinical presentation	Diagnosis	Inguinal hernia	Managed by
1	Rahim et al. [8], 1980		M	Ulceration and discharge, scrotum	ECF-inguinal area	Present	Exploratory laparotomy
2	Rao et al. [9], 1980	<1	M	Ulceration and discharge, scrotum	ECF-inguinal area	Present	Exploratory laparotomy
3	Rao et al. [9], 1980		M	Iatrogenic fecal fistula, scrotum	ECF-inguinal area	Present	Exploratory laparotomy
4	Kapoor et al. [10], 1991	<1	M	Ulceration and discharge, scrotum	ECF-inguinal area	Present	Exploratory laparotomy
5	Rattan et al. [6], 1998	<1	M	Ulceration and discharge, scrotum	ECF-inguinal area	Present	Exploratory laparotomy
6	Kasat et al. [11], 2000	<1	M	Ulceration and discharge, scrotum	ECF-inguinal area	Present	Exploratory laparotomy
7	Ameh et al. [12], 2002	<1		Fecal fistula, scrotum	ECF-inguinal area	Present	Exploratory laparotomy
8	Ameh et al. [12], 2002	<1		Fecal fistula, scrotum	ECF-inguinal area	Present	Exploratory laparotomy
9	Samad and Sheikh [13], 2005	25	M	Ulceration and discharge, scrotum	ECF-inguinal area	Present	Exploratory laparotomy
10	Koshariya et al. [14], 2006		M	Ulceration and discharge, scrotum	ECF-inguinal area	Present	Exploratory laparotomy
11	Sowande et al. [15], 2006	<1	M	Ulceration and discharge, scrotum	ECF-inguinal area	Present	Exploratory laparotomy
12	Ghritlaharey et al. [7], 2007	<1	M	Ulceration and discharge, scrotum	ECF-inguinal area	Present	Exploratory laparotomy
13	Sheikh et al. [16], 2009	42	M	Ulceration and discharge, scrotum	ECF-inguinal area	Present	Exploratory laparotomy
14	Chirdan et al. [17], 2010	21	M	Ulceration and discharge, scrotum	ECF-inguinal area	Present	Exploratory laparotomy
15	Saravana et al. [18], 2010	26	M	Ulceration and discharge, scrotum	ECF-inguinal area	Present	Exploratory laparotomy
16	Ohene-Yeboah [19], 2011		M	Ulceration and discharge, scrotum	ECF-inguinal area	Present	Exploratory laparotomy
17	Ohene-Yeboah [19], 2011		M	Ulceration and discharge, scrotum	ECF-inguinal area	Present	Exploratory laparotomy
18	Ezomike et al. [20], 2012	<1	M	Fecal fistula, scrotum	ECF-inguinal area	Present	Exploratory laparotomy
19	Bhasin et al. [21], 2013	65	M	Iatrogenic fecal fistula, scrotum	ECF-inguinal area	Present	Exploratory laparotomy
20	Bhasin et al. [21], 2013	40	M	Ulceration and discharge, scrotum	ECF-inguinal area	Present	Exploratory laparotomy
21	Malik et al. [1], 2014	70	M	Ulceration and discharge, scrotum	ECF-inguinal area	Present	Exploratory laparotomy
22	Ahi et al. [22], 2015	62	M	Fecal discharge, inguinal region	ECF-inguinal area	Present	Exploratory laparotomy
23	Degheili et al. [23], 2015	75	M	Post-TAPP inflammatory swelling scrotum	Fecal abscess-scrotum	Present	Exploratory laparotomy
24	Ota et al. [24], 2015	79	M	Swollen and inflamed, scrotum	Fecal abscess-scrotum	Present	Patient refused for exploratory laparotomy
25	Panagidis et al. [25], 2015	<1	M	Ulceration and discharge, scrotum	ECF-inguinal area	Present	Exploratory laparotomy
26	Ajape et al. [26], 2016	28	M	Ulceration and discharge, scrotum	ECF-inguinal area	Present	Exploratory laparotomy
27	Arora [27], 2016	35	M	Fecal fistula, scrotum	ECF-inguinal area	Present	Exploratory laparotomy
28	Elenwo et al. [28], 2016	61	F	Ulceration and discharge, scrotum	ECF-labial	Present	Exploratory laparotomy
29	Hajong et al. [29], 2017	53	M	Fecal discharge from right groin	ECF-inguinal area	Present	Exploratory laparotomy
30	Raj et al. [30], 2018	32	M	Ulceration and discharge, scrotum	ECF-inguinal area	Present	Exploratory laparotomy
31	Amoako et al. [5], 2018	32	M	Ulceration and discharge, scrotum	ECF-inguinal area	Present	Exploratory laparotomy
32	Omran et al. [31], 2019	<1	M	Swollen and inflamed, scrotum	Fecal abscess-scrotum	Present	Exploratory laparotomy
33	Asghar et al. [32], 2020	60	M	Ulceration and discharge, scrotum	ECF-inguinal area	Present	Patient refused for exploratory laparotomy
34	Bahadur et al. (present case), 2021	20	M	Swollen and inflamed, scrotum	Fecal abscess-scrotum	Absent	Drainage of the fecal scrotal abscess

## Data Availability

All the datasets on which the manuscript relies are available in the Department of Surgery, Dr. Hedgewar Arogya Sansthan.

## Abbreviations

SPC: Spontaneous perforation of the colon
USG: Ultrasonography
CT: Computed tomography
MRI: Magnetic resonance imaging
I&D: Incision & drainage
ECF: Enterocutaneous fistula.
